# GLP-1 receptor agonists—a potential therapy for chronic kidney disease?

**DOI:** 10.1042/CS20261312

**Published:** 2026-10-01

**Authors:** Jan Boeckhaus, John A. Sayer, Holly Mabillard

**Affiliations:** 1 Department of Intensive Care, King's College London, Guy's and St Thomas' Hospital, London, U.K.; 2 Department of Nephrology and Rheumatology, University Medical Center Göttingen, Goettingen, Germany; 3 Renal Services, Newcastle upon Tyne Hospitals NHS Foundation Trust, Newcastle upon Tyne, U.K.; 4 NIHR Newcastle Biomedical Research Centre, Newcastle upon Tyne, U.K.; 5 Biosciences Institute, Faculty of Medical Sciences, Newcastle University, Central Parkway, Newcastle upon Tyne, U.K.; 6 Translational and Clinical Research Institute, Faculty of Medical Sciences, Newcastle University, Central Parkway, Newcastle upon Tyne, U.K.

**Keywords:** cardiovascular physiologyscular, chronic kidney disease, glucagon-like peptide-1, nephrology

## Abstract

Chronic kidney disease (CKD) represents a significant health burden worldwide, characterized by a gradual decline in kidney function and increased risk of cardiovascular events. Current therapeutic strategies like inhibition of the renin-angiotensin-aldosterone system or sodium-glucose cotransporter 2 inhibitors (SGLT2i), while beneficial, often fail to fully halt CKD progression, and a significant residual risk of disease progression persists. Glucagon-like peptide-1 receptor agonists (GLP-1 RAs), initially developed for the treatment of type 2 diabetes (T2D), have emerged as promising agents with pleiotropic effects extending beyond glycemic control and weight loss. Evidence from major cardiovascular outcome trials and dedicated CKD studies demonstrates that GLP-1 RAs can confer significant cardiovascular and nephroprotective benefits in patients with T2D and CKD. These benefits include reductions in major adverse cardiovascular events, slowing of kidney disease progression (as measured by estimated glomerular filtration rate (eGFR) decline), and decreased albuminuria. The mechanisms underlying these effects are complex and involve multiple pathways, including anti-inflammatory, anti-fibrotic, and antioxidative actions, as well as improvements in hemodynamic parameters. We provide a comprehensive overview of the current clinical and preclinical evidence supporting the use of GLP-1 RAs in CKD. We discuss the proposed mechanisms of action by which GLP-1 RAs exert their nephroprotective effects and explore the potential for these agents to improve kidney outcomes across a broad spectrum of patients with CKD and critically evaluate the potential risks and safety profile associated with GLP-1 RA use.

## Introduction

The global health impact of chronic kidney disease (CKD) remains substantial, with frequent progression to kidney failure and a consequent rise in morbidity and mortality [1]. Despite advancements in treatment, CKD is predicted to rise to the fifth-highest cause of death in 2050 worldwide [2]. CKD is defined by the gradual attrition of kidney function. Pathophysiologically, the “remnant nephron” hypothesis describes an early phase of hyperfiltration that, while temporarily sustaining function, eventually leads to progressive glomerulosclerosis [3]. This transition from adaptation to injury is mediated by a variety of molecular drivers that catalyze the loss of the kidney architecture.

Progressive CKD is characterized by tubulointerstitial fibrosis, driven by a synergistic interplay between oxidative stress and chronic inflammation [4,5]. The accumulation of reactive oxygen species (ROS) triggers pro-inflammatory cascades and growth factor signaling, precipitating cellular injury and functional decline. Key cytokines like tumor necrosis factor-α (TNF-α) and interleukin-6 (IL-6) exacerbate tissue damage, whereas transforming growth factor-β (TGF-β) drives fibroblast-to-myofibroblast transformation, the primary cause of renal scarring and parenchymal loss [6,7]. Furthermore, pathological overactivation of the renin-angiotensin-aldosterone system (RAAS) is a primary driver of kidney disease progression. The resulting rise in angiotensin II drives damage through two distinct pathways. Hemodynamically, it causes systemic and efferent arteriolar vasoconstriction, leading to hypertension; non-hemodynamically, it acts as a pro-inflammatory and pro-fibrotic cytokine that accelerates glomerular scarring and renal tissue damage [8,9]. CKD significantly increases the risk of cardiovascular events. Hence, there is a pressing need for treatments that can effectively manage both kidney and cardiovascular complications [10].

## The evolving therapeutic landscape of CKD: the emerging role of GLP-1 RAs

For decades, the management of CKD largely focused on controlling risk factors such as hypertension and diabetes. The discovery of angiotensin-converting enzyme inhibitors and angiotensin II type 1 receptor blockers established a new standard of care for CKD by effectively slowing disease progression through modulation of the RAAS [11–13]. The past decade has witnessed the emergence of novel classes of medications that have redefined the therapeutic landscape of CKD. Sodium-glucose cotransporter 2 inhibitors (SGLT2i), initially developed for the treatment of type 2 diabetes (T2D), have demonstrated remarkable benefits in reducing the risk of cardiovascular events and slowing the progression of CKD [14]. By inhibiting the sodium-glucose cotransporter 2 in the proximal tubule, SGLT2i reduce albuminuria and slow the progression of kidney disease in patients with and without T2D [15–17]. While the precise mechanisms underlying the nephroprotective effects of SGLT2i remain uncertain, proposed pathways include reduction in renal ischemia as well as inflammation and fibrosis suppression [18–20]. Furthermore, finerenone, a selective, non-steroidal mineralocorticoid receptor antagonist, has also shown to be nephroprotective in diabetic CKD due to its combined anti-fibrotic, anti-inflammatory, and anti-oxidative effects [21]. The recent phase III clinical trials FIGARO-DKD (NCT02545049) and FIDELIO-DKD (NCT02540993) have demonstrated finerenone’s potential in lowering the risk of kidney disease progression and incidence of cardiovascular events in patients with diabetic CKD [22,23]. The clinical utility of finerenone is currently being expanded: Results from the FIND-CKD trial (NCT05047263) demonstrated that finerenone (10 or 20 mg daily), added to background RAAS inhibition in 1584 adults with non-diabetic CKD, significantly slowed estimated glomerular filtration rate (eGFR) loss and lowered the risk of a composite kidney–cardiovascular outcome compared with placebo [24]. Furthermore, the FIONA (NCT05196035) and FIONA OLE trials (NCT05457283) assess the safety and efficacy of finerenone in children and adolescents with CKD [25]. In addition, endothelin receptor antagonists have further expanded the therapeutic landscape. Results from the SONAR trial with atrasentan (NCT01858532), alongside the PROTECT (NCT03762850) and ALIGN (NCT04573478) trials with sparsentan and atrasentan in IgA nephropathy, have demonstrated that targeting the endothelin pathway is effective in reducing albuminuria [26–28].

Despite these advances in CKD management, a substantial residual risk remains, with many patients experiencing disease progression and adverse cardiovascular events. This necessitates the development of novel therapeutic strategies to improve patient outcomes. Glucagon-like peptide-1 receptor agonists (GLP-1 RAs), initially developed for T2D, have emerged as promising agents with potential nephroprotective effects (Figure 1). By modulating various metabolic pathways, including glucose metabolism, insulin secretion, and potentially influencing inflammation and fibrosis, GLP-1 RAs may offer substantial benefits for individuals with CKD. The discovery of GLP-1 RAs has an interesting historical context. The identification of exendin-4, a potent GLP-1 RA, in the venom of the Gila monster (*Heloderma suspectum*), a lizard native to the southwestern United States and Mexico, played a key role in their development [29]. The evolutionary role of glucagon-like peptide-1 (GLP-1) in the Gila monster is linked to metabolic adaptation in harsh environments through enhanced insulin secretion and delayed gastric emptying. Exendin-4, a GLP-1 RA found in Gila monster saliva, mimics human GLP-1 but with enhanced stability and a longer duration of action, paving the way for the development of GLP-1 receptor-based therapies [30]. While initially established for glycemic management in T2D, emerging evidence indicates that GLP-1 RAs confer cardiovascular benefits regardless of diabetic status; furthermore, recent clinical and preclinical findings support a nephroprotective role (Table 1). Therefore, in adults with T2D and established cardiovascular disease (CVD) or at high CV risk, the 2022 Kidney Disease: Improving Global Outcomes (KDIGO) guidelines recommend long-acting GLP-1 RAs with demonstrated CV benefits as a preferred second-line therapy for glycemic control and CV risk reduction, particularly when metformin and SGLT2i therapy is insufficient or contraindicated [31]. These agents exert their effects by mimicking endogenous GLP-1, thereby stimulating insulin secretion, suppressing glucagon release, and slowing gastric emptying, ultimately leading to weight loss and improved glycemic control [32]. Beyond their proven efficacy in diabetic kidney disease, GLP-1 RAs represent a promising frontier for CKD more broadly.

**Figure 1:**
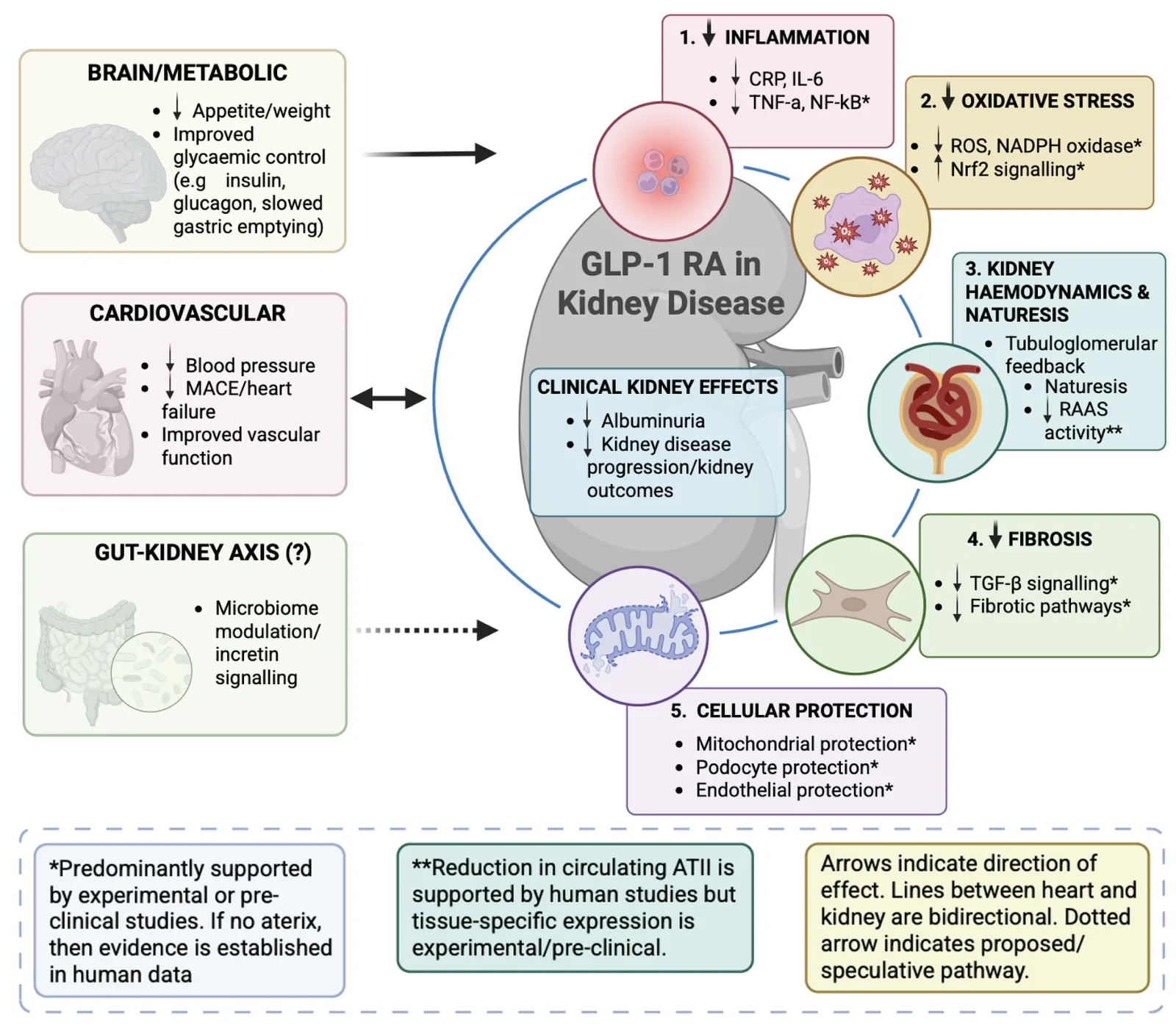
Systems-level mechanisms underlying the renal protective effects of GLP-1 receptor agonists in CKD This schematic summarizes the multi-system and renal-specific mechanisms through which GLP-1 RAs may confer nephroprotection in CKD, while distinguishing clinically demonstrated effects from mechanisms supported predominantly by experimental or preclinical evidence. Progressive CKD is characterized by inflammation, oxidative stress, endothelial dysfunction, and fibrosis, which together contribute to albuminuria, podocyte injury, and progressive loss of kidney function. Clinically demonstrated kidney effects of GLP-1 RAs include reductions in albuminuria and kidney disease progression/composite kidney outcomes. GLP-1 RAs attenuate inflammation (**1**), with clinical studies demonstrating reductions in circulating inflammatory markers, including CRP and IL-6. Experimental studies additionally support down-regulation of pro-inflammatory pathways involving TNF-α and NF-κB, together with modulation of macrophage polarization towards an anti-inflammatory M2 phenotype. GLP-1 RAs may inhibit oxidative stress (**2**) by reducing NAD(P)H oxidase activity via cAMP–PKA signaling, resulting in lower ROS levels in glomerular and tubular compartments. In parallel, activation of the Nrf2 pathway may enhance endogenous antioxidant defenses. These oxidative stress pathways are predominantly supported by experimental and preclinical studies. Renal hemodynamic and tubular effects represent a key nephrology-specific mechanism (**3**). GLP-1 RAs promote natriuresis through inhibition of the Na^+^/H^+^ exchanger 3 in the proximal tubule, reducing proximal tubular sodium reabsorption and increasing distal sodium delivery. This may activate tubuloglomerular feedback, thereby reducing intraglomerular pressure. GLP-1 RAs may also modulate the RAAS, with human mechanistic studies demonstrating reductions in circulating angiotensin II and experimental studies supporting down-regulation of tissue-level angiotensin II. Together, these hemodynamic and tubular effects may contribute to reductions in albuminuria and slowing of eGFR decline. Fibrotic responses (**4**) may be suppressed through inhibition of TGF-β signaling, with experimental studies demonstrating reductions in fibronectin and collagen deposition and modulation of extracellular matrix turnover. GLP-1 RAs may additionally exert direct cellular protective effects (**5**). These include preservation of podocyte integrity through inhibition of TNF-α/NF-κB signaling and modulation of AMP-activated protein kinase pathways, and protection of endothelial function, including maintenance of endothelial glycocalyx and vascular integrity, which may support glomerular filtration barrier stability. Experimental studies also suggest suppression of endothelial inflammatory pathways, including endothelin-1 and receptor for advanced glycation end-products (RAGE) signaling. Emerging cellular mechanisms include mitochondrial protection, characterized by preservation of mitochondrial membrane potential and integrity, reduced ROS generation, and attenuation of mitochondrial apoptosis. Regulation of autophagy pathways may additionally contribute to cellular homeostasis and protein quality control. Evidence supporting these mitochondrial, podocyte, endothelial, antifibrotic, and autophagy-related mechanisms is predominantly derived from experimental or preclinical studies, with limited human mechanistic data and no established evidence that these pathways mediate the observed clinical kidney benefits. Beyond the kidney, GLP-1 RAs act across interconnected organ systems. Central nervous system and metabolic effects include regulation of appetite and body weight, increased insulin secretion, suppression of glucagon, delayed gastric emptying, and improved glycemic control. These systemic metabolic effects may indirectly reduce kidney injury. Cardiovascular effects include reductions in blood pressure, HF events and MACE, improvements in vascular endothelial function and lipid profiles, and potential anti-atherosclerotic effects. Bidirectional interactions within the cardiorenal axis may therefore contribute to overall kidney protection. Gut-derived signaling through the gut–kidney axis, including modulation of the intestinal microbiome and incretin signaling, represents an additional proposed pathway linking extra-renal GLP-1 RA effects with kidney outcomes. The figure distinguishes the strength of evidence supporting these pathways. Clinically demonstrated kidney effects are shown separately from proposed biological mechanisms. An asterisk (*) denotes mechanisms predominantly supported by experimental or preclinical studies. A double asterisk (**) denotes pathways for which human mechanistic evidence supports reductions in circulating angiotensin II, while evidence for tissue-level angiotensin II/RAAS modulation remains experimental or preclinical. Dotted arrows indicate proposed or speculative inter-organ pathways, and the heart–kidney relationship is shown as bidirectional. Figure created using BioRender.

**Table 1:** Summary of trials using GLP-1 RAs

Trial	Year	Objective	Design	Population	Primary outcome	Results
**SUSTAIN-6**	2016	Investigate semaglutide’s effect on CVD risk in T2D patients	Randomized, double-blind, placebo-controlled	Patients aged 50+ with T2D, established CVD, heart failure, or CKD (stage III+), or aged 60+ with CVD risk factors	Composite of CV death, non-fatal MI, or non-fatal stroke	Semaglutide reduced the composite outcome significantly (6.6% versus 8.9% in placebo; HR: 0.74, CI: 0.58–0.95; *P* < 0.001). Fewer serious adverse events occurred, but more patients discontinued due to gastrointestinal side effects.
**LEADER**	2016	Investigate liraglutide’s effect on CV outcomes in T2D patients	Randomized, double-blind, placebo-controlled	Patients with T2D and high CV risk	Composite of death from cardiovascular causes, non-fatal MI, or non-fatal stroke	Liraglutide reduced CV events (13.0% versus 14.9% in placebo; HR: 0.87, CI: 0.78–0.97; *P* < 0.001 for non-inferiority; *P* = 0.01 for superiority).
**Harmony Outcomes**	2018	Investigate albiglutide’s effect on CV outcomes in T2D patients	Randomized, double-blind, placebo-controlled	Patients aged 40+ with T2D and established CVD	Composite of CV death, MI, or stroke	Albiglutide reduced CV events (7% versus 9% in placebo; HR: 0.78, CI: 0.68–0.90; P < 0.0001 for non-inferiority; P = 0.0006 for superiority).
**REWIND**	2019	Investigate dulaglutide’s effect on major CV events in T2D patients	Randomized, double-blind, placebo-controlled	Patients aged 50+ with T2D, with prior CV event or risk factors	First occurrence of non-fatal MI, non-fatal stroke, or CV death	Dulaglutide reduced major CV events (12.0% versus 13.4% in placebo; HR: 0.88, CI: 0.79–0.99; *P* = 0.026). Gastrointestinal events more frequent with dulaglutide.
**AMPLITUDE-O**	2021	Investigate efpeglenatide’s effect on CV and CKD outcomes in T2D patients	Randomized, double-blind, placebo-controlled	Patients with T2D and history of CVD or eGFR 25.0-59.9 ml/min/1.73 m^2^	CV events and composite renal outcomes	Efpeglenatide reduced MACE and renal outcomes (13.0% versus 18.4% in placebo). Gastrointestinal side effects were higher with efpeglenatide.
**AWARD-PEDS**	2022	Investigate dulaglutide’s efficacy and safety in adolescents with T2D	Randomized, double-blind, placebo-controlled	Adolescents aged 10 to <18 with BMI above the 85th percentile	Change in HbA1c	Dulaglutide reduced HbA1c levels significantly compared with placebo (−0.6% to −0.9%). Higher incidence of gastrointestinal adverse events in dulaglutide group.
**SELECT**	2023	Investigate semaglutide’s effect on CV outcomes in overweight/obese individuals without diabetes	Randomized, double-blind, placebo-controlled	Overweight/obese patients aged 45+ with established CVD	Composite of CV death, non-fatal MI, or non-fatal stroke	Semaglutide reduced CV events (6.5% versus 8.0% in placebo; HR: 0.80, CI: 0.72–0.90; *P* < 0.001). More discontinuation in semaglutide group due to side effects.
**FLOW**	2024	Investigate semaglutide’s effect on kidney outcomes in CKD patients with T2D	Randomized, double-blind, multinational phase 3b trial	Patients with CKD and T2D	Composite of eGFR decline, kidney failure, or death from kidney/CV causes	Semaglutide reduced primary outcome events by 24%. Slower eGFR decline observed, but more frequent discontinuation due to gastrointestinal side effects.
**SOUL**	2025	Investigate cardiovascular efficacy of oral semaglutide in T2D and atherosclerotic CVD, CKD, or both	Randomized, double-blind, multinational, event-driven phase 3b trial	Patients aged at least 50 years, with T2D and established atherosclerotic CVD and/or CKD	Composite of death from cardiovascular causes, nonfatal myocardial infarction, or non-fatal stroke	Semaglutide significantly reduced the risk of MACE by 14% (HR: 0.86, CI: 0.77–0.96; *P* = 0.006) compared with placebo.

Abbreviations: BMI, body mass index; CI, confidence interval; CKD, chronic kidney disease; CV, cardiovascular; CVD, cardiovascular disease; eGFR, estimated glomerular filtration rate; GLP-1 RA, glucagon-like peptide-1 receptor agonist; HbA1c, glycated hemoglobin; HR, hazard ratio; MACE, major adverse cardiovascular events; MI, myocardial infarction; T2D, type 2 diabetes.

## GLP-1 receptor agonists and cardiorenal protection: from albuminuria reduction to major adverse cardiovascular events (MACE) prevention

### GLP-1 RAs and albuminuria: early signals of nephroprotection

Lowering albuminuria, specifically as measured by the urinary albumin-to-creatinine ratio (UACR), serves as a critical early indicator of attenuated kidney function decline and a potent predictor of cardiovascular risk [33,34]. However, the persistence of residual albuminuria despite standard-of-care treatment is associated with accelerated kidney disease progression and adverse renal events, underscoring the urgent need for additional interventions to improve long-term outcomes [35,36]. Several studies have investigated the impact of GLP-1 RAs on albuminuria in various patient populations. In the AWARD-7 trial, once-weekly dulaglutide demonstrated non-inferiority to insulin glargine in glycemic control among patients with T2D and CKD stages G3–4. Over 52 weeks, dulaglutide was associated with a significantly smaller decline in eGFR compared with insulin glargine, suggesting a renal protective effect despite UACR differences not being significantly different. Furthermore, while dulaglutide increased gastrointestinal adverse events, it reduced the rate of symptomatic hypoglycemia compared with basal insulin therapy [37]. In the SCALE trial, liraglutide led to a reduction in UACR in a dose-dependent manner [38]. A pooled analysis of the SUSTAIN-6 and LEADER trials demonstrated that semaglutide and liraglutide significantly reduced albuminuria from baseline to two years post-randomization compared with placebo [39]. In addition, a pre-specified analysis of the SELECT trial showed a reduction of albuminuria with a net treatment benefit of −10.7% (95% confidence interval (CI) −13.2, −8.2), suggesting a beneficial kidney effect of once-weekly semaglutide in patients with overweight or obesity and established CVD without diabetes [40]. Furthermore, an exploratory analysis of the STEP 1, 2, and 3 trials demonstrated that semaglutide improved albuminuria in adults with overweight or obesity and T2D [41]. A post hoc analysis of the LEADER trial demonstrated that early reductions in albuminuria with liraglutide were directly associated with a lower risk of subsequent cardiovascular and renal events [42]. Recently, the SMART trial demonstrated that once-weekly semaglutide reduced albuminuria by 52.1% (95% CI: −65.5, −33.4; *P* < 0.0001) compared with placebo in adults with CKD (eGFR ≥25 ml/min/1.73 m^2^ and UACR ≥30 and <3500 mg/g creatinine) and a body mass index (BMI) of ≥27 kg/m^2^. Chronic glomerulonephritis and hypertensive CKD were the most common CKD etiologies in the SMART trial [43]. In a meta-analysis of 15 RCTs involving over 14 000 participants, superior UACR reductions by tirzepatide, a dual agonist of the GLP-1 receptor and the glucose-dependent insulinotropic polypeptide receptor, compared with both placebo and insulin were demonstrated, with particularly pronounced effects in patients with T2D (mean difference −33.3% versus −7.9% in obesity alone) [44].

### Mitigating cardiovascular risk: impact on MACE

Beyond their role in slowing diabetic kidney disease progression, GLP-1 RAs have demonstrated robust cardioprotective effects [45]. Evidence from landmark cardiovascular outcome trials (specifically involving albiglutide, efpeglenatide, dulaglutide, liraglutide, and weekly semaglutide) confirms a significant reduction in MACE among patients with T2D and high cardiovascular risk, alongside further cardiovascular benefits such as symptomatic improvement in obesity-related heart failure [46,47,48,49,50,51].

SUSTAIN-6 evaluated the CV effects of once-weekly semaglutide (0.5 mg or 1.0 mg) in patients with T2D who were either aged ≥50 years with established CVD or CKD, or aged ≥60 years with cardiovascular risk factors alone. Semaglutide significantly reduced the primary composite CV outcome (CV death, non-fatal myocardial infarction, or non-fatal stroke) compared with placebo (HR: 0.74, 95% CI: 0.58–0.95; *P* < 0.001 for non-inferiority), despite a higher rate of treatment discontinuation due to primarily gastrointestinal side effects. Serious adverse events (SAEs) were less frequent with semaglutide [46].

In AMPLITUDE-O, adults (≥18 years) with T2D and either established CVD or an eGFR of 25–59.9 ml/min/1.73 m^2^ and at least one additional CV risk factor were randomized to weekly efpeglenatide (4 mg or 6 mg) or placebo. Efpeglenatide significantly reduced both MACE and a composite renal outcome (kidney function decline or macroalbuminuria; 13.0% versus 18.4% with placebo), but was associated with a higher incidence of gastrointestinal adverse events (diarrhea and nausea) [47].

The REWIND trial evaluated the effect of dulaglutide (1.5 mg/week) on MACE (non-fatal myocardial infarction, non-fatal stroke, or cardiovascular death) in a broad population of patients aged at least 50 years with T2D and either a previous cardiovascular event or cardiovascular risk factors. During a median follow-up of 5.4 years, dulaglutide significantly reduced MACE compared with placebo (HR: 0.88, 95% CI: 0.79–0.99; *P* = 0.026), but was associated with a higher incidence of gastrointestinal adverse events (47.4% versus 34.1%; *P* < 0.0001) [48].

In the Harmony Outcomes trial, once-weekly albiglutide (30–50 mg) significantly reduced the primary CV outcome (CV death, myocardial infarction, or stroke) compared with placebo (7% versus 9%; HR: 0.78, 95% CI: 0.68–0.90; *P* = 0.0006 for superiority) in patients ≥40 years with T2D and established CVD [49].

In the LEADER trial, liraglutide significantly reduced the primary composite CV outcome (CV death, non-fatal myocardial infarction, or non-fatal stroke) compared with placebo (13.0% versus 14.9%; HR: 0.87, 95% CI: 0.78–0.97; *P* = 0.01 for superiority) in patients with T2D and high CV risk. Gastrointestinal events were the most common reason for treatment discontinuation with liraglutide [51].

A post hoc analysis of the SURPASS-4 trial, a phase 3 study in adults with T2D and CVD or a high risk of cardiovascular events, found that once-weekly tirzepatide significantly reduced the composite kidney outcome (≥40% eGFR decline, end-stage kidney disease, death owing to kidney failure, or new macroalbuminuria) compared with once-daily insulin glargine (HR: 0.58 [95% CI: 0.43–0.80]). Tirzepatide also slowed eGFR decline and reduced UACR more effectively than insulin glargine [52].

In the AWARD-PEDS trial, once-weekly dulaglutide (0.75 mg or 1.5 mg) significantly reduced glycated hemoglobin (HbA1c) levels at 26 weeks compared with placebo (−0.6% and −0.9% versus +0.6%, respectively; both *P* < 0.001) in adolescents (10 to <18 years) with T2D and a BMI above the 85th percentile. Dulaglutide was associated with a higher incidence of gastrointestinal adverse events, reported to be consistent with the adult safety profile [53].

In the FLOW trial, which enrolled a high-risk population of patients with T2D and CKD (defined as an eGFR of 50–75 ml/min/1.73 m^2^ and a UACR of >300 and <5000 mg/g or an eGFR of 25–49 ml/min/1.73 m^2^ and a UACR of >100 and <5000 mg/g), once-weekly semaglutide (1.0 mg) significantly reduced the primary composite kidney outcome (kidney failure, ≥50% eGFR reduction, or kidney-related or cardiovascular death) by 24% compared with placebo (HR: 0.76, 95% CI: 0.66–0.88; *P* = 0.0003; NNT = 20). The trial was terminated early for efficacy. Semaglutide also reduced UACR more effectively than placebo at 104 weeks (40% versus 12%). While patients with semaglutide treatment had a slower eGFR decline (−2.19 versus −3.36 ml/min/1.73 m^2^ per year), it led to more frequent discontinuation mainly due to gastrointestinal side effects. SAEs were reported less frequently in participants in the semaglutide group compared with the placebo group (49.6% versus 53.8%) [54,55]. While the FLOW trial was not specifically powered to evaluate kidney failure, aggregate data from large-scale clinical trials suggest that GLP-1 RAs may attenuate the risk of kidney failure by approximately 16% among patients with T2D [56].

In the SOUL trial, once-daily oral semaglutide significantly reduced the risk of MACE by 14% compared with placebo in patients aged at least 50 years, with T2D and established atherosclerotic CVD and/or CKD (HR: 0.86; 95% CI: 0.77-0.96; *P* = 0.006). While the drug demonstrated superior cardiovascular efficacy over a median follow-up of 49.5 months, no significant differences were observed in the confirmatory secondary outcomes, which included major kidney disease events. The incidence of SAEs remained comparable to placebo [57].

In the SELECT trial, semaglutide (2.4 mg/week) significantly reduced the primary composite CV outcome (CV death, non-fatal myocardial infarction, or non-fatal stroke) compared with placebo (6.5% versus 8.0%; HR: 0.80, 95% CI: 0.72–0.90; *P* < 0.001) in overweight or obese patients (BMI ≥27 kg/m^2^) aged ≥45 years with established CVD. Discontinuation due to adverse events was more frequent with semaglutide (16.6% versus 8.2%) [58]. Furthermore, semaglutide reduced the incidence of a pre-specified composite kidney endpoint (kidney disease death, kidney replacement therapy initiation, persistent eGFR <15 ml/min/1.73 m^2^, ≥50% eGFR reduction, or onset of persistent macroalbuminuria) compared with placebo (1.8% versus 2.2%; HR: 0.78, 95% CI: 0.63–0.96; *P* = 0.02). Semaglutide also improved eGFR at 104 weeks overall (0.75 ml/min/1.73 m^2^, 95% CI: 0.43–1.06; *P* < 0.001) and more substantially in those with baseline eGFR <60 ml/min/1.73 m^2^ (2.19 ml/min/1.73 m^2^, 95% CI: 1.00–3.38; *P* < 0.001), suggesting a kidney benefit in overweight or obese patients without diabetes [40]. Incorporating recent data from the SOUL and FLOW trials, a meta-analysis of over 71 000 individuals with T2D found that long-acting GLP-1 RAs provide a robust 14% reduction in MACE and a 17% reduction in a composite kidney outcome. Notably, these benefits were consistent across both injectable and oral formulations [59].

While these landmark cardiovascular outcome trials have demonstrated the efficacy of GLP-1 RAs, recent real-world data emphasize that these benefits are highly contingent upon treatment persistence. Evidence from large-scale target trial emulations suggests that the cardiovascular benefits of GLP-1 RAs are duration-dependent, as treatment interruptions or early discontinuation may progressively erode this protective effect, potentially leading to an increased risk of adverse cardiovascular events compared with sustained use [60].

### Trial limitations and residual uncertainty

Some limitations temper the interpretation of this clinical evidence. Composite kidney endpoints are defined inconsistently across trials: thresholds for eGFR decline differ (≥40% versus ≥50%), some composites incorporate cardiovascular death while others do not, and several include new-onset macroalbuminuria. Evidence in non-diabetic CKD relies primarily on surrogate outcomes; the SMART trial randomized 101 participants over 24 weeks with percentage change in UACR as the primary endpoint, and whether albuminuria reduction translates to preserved kidney function in this population remains to be further investigated. In many trial protocols, patients with severely impaired kidney function and individuals requiring dialysis were excluded, and evidence in these groups remains observational. Critically, many trials enrolled participants prior to the widespread clinical adoption of SGLT2i and non-steroidal mineralocorticoid receptor antagonists; thus, whether GLP-1 RAs confer incremental protection alongside these therapies remains to be further established. Preliminary evidence comes from the FLOW trial, in which 15.6% of participants were taking an SGLT2i at baseline [61]. Among these, the primary outcome occurred in 41 of 277 participants assigned to semaglutide and 38 of 273 assigned to placebo (HR: 1.07, 95% CI: 0.69–1.67). Among those not taking an SGLT2i, the corresponding figures were 290 of 1490 and 372 of 1493 (HR: 0.73, 95% CI: 0.63–0.85) without a statistically significant interaction (*P* = 0.109) [61]. Finally, as this drug class is heterogeneous, individual trial results should not be assumed to generalize across all GLP-1 RAs.

## Beyond systemic risk factors: the pleiotropic effects of GLP-1 RAs

The pleiotropic effects of GLP-1 RAs can be conceptualized across interconnected biological systems, including central nervous system appetite regulation, intestinal microbiome modulation, vascular endothelial function, and direct renal cellular signaling.

Combined analyses of cardiovascular outcome trials suggested nephroprotective potential for GLP-1 RAs in T2D, particularly for semaglutide, efpeglenatide, dulaglutide, and liraglutide, through a reduced risk of composite kidney outcomes (macroalbuminuria, significant decline in kidney function, kidney failure, and kidney disease death). However, until the FLOW trial, this benefit appeared largely driven by a reduction in persistent macroalbuminuria [62,63]. The recent SMART trial has now demonstrated the potential of GLP-1 RAs to reduce UACR in non-diabetic CKD. The renoprotective mechanisms of GLP-1 RAs appear to be multifactorial. Beyond its initial identification in pancreatic β cells, the GLP-1 receptor (GLP-1R) transduces pleiotropic effects across multiple organs, including the heart, vasculature, and kidneys [64,65]. Whether the renal effects result from direct GLP-1R activation was a topic of discussion [66]. While earlier reports suggested broader intrarenal distribution, studies utilizing validated monoclonal antibodies and transcriptomic atlases localize GLP-1R primarily to preglomerular vascular smooth muscle and juxtaglomerular cells [64,67].

A growing body of evidence suggests that GLP-1 signaling protects against endothelial injury/dysfunction, reduces oxidative stress, and mitigates inflammation, but the precise mechanisms by which GLP-1 RAs exert kidney-protective effects remain under investigation (Table 2) [68]. GLP-1 and other intestinal peptides serve as signaling mediators across several interorgan pathways, including the gut–kidney axis [69]. Although improved glycemic control, weight loss, lipids, and reduced blood pressure likely contribute to these benefits, mediation analyses suggest they do not fully account for the observed renoprotection, implying the involvement of additional mechanisms [70–72].

**Table 2:** Kidney-protective effects and proposed mechanisms of GLP-1 RAs, categorized by the level of supporting evidence synthesized in this review

Effect or proposed mechanism	Evidence	Uncertainty
**Clinical studies**		
Reduction in composite kidney outcomes (T2D)	Meta-analyses of cardiovascular outcome trials; dedicated outcome trial (FLOW)	Pre-FLOW, benefit was driven largely by albuminuria components
Reduction in albuminuria (diabetic and non-diabetic CKD)	Randomized trials (FLOW, SMART)	No hard kidney-outcome trial in non-diabetic CKD yet
Reduction in systemic inflammation (hsCRP, IL-6)	Meta-analyses of RCTs; pooled semaglutide-trial analysis (SUSTAIN/PIONEER)	hsCRP reduction not significant in the CKD subgroup (PIONEER 5); causal role in kidney protection under investigation
**Human mechanistic studies**		
Natriuresis and reduced proximal tubular sodium reabsorption	Healthy/obese volunteers; small mechanistic studies	Not demonstrated in CKD
Reduction in circulating angiotensin II	Small human studies	Not studied in dedicated CKD populations
**Animal models**		
Down-regulation of tissue-level angiotensin II	Rodent study with GLP-1 receptor blockade	No human confirmation
cAMP-PKA signaling and NAD(P)H oxidase inhibition	*Glp1r* knockout mice with pharmacological dissection	Not confirmed in human tissue
Antifibrotic effect via TGF-β suppression	Murine ureteral-obstruction model	No human confirmation
Mitochondrial protection and reduced reactive oxygen species	Rodent and cell-based models with pharmacological dissection; single human leukocyte study	Human data are non-renal; mediation of functional benefit untested
Podocyte protection (TNF-α/NF-κB)	Obese and db/db mouse models; cultured-podocyte support	No human confirmation
**Cell-based and *in vitro***		
M2 macrophage polarization	*In vitro* human macrophages	Not demonstrated *in vivo* in kidney
Endothelial anti-inflammatory effects (endothelin-1 suppression, RAGE down-regulation)	*In vitro* endothelial cells	Not demonstrated *in vivo* in kidney

Abbreviations: cAMP, cyclic adenosine monophosphate; CKD, chronic kidney disease; GLP-1 RA, glucagon-like peptide-1 receptor agonist; *Glp1r*, glucagon-like peptide-1 receptor gene; hsCRP, high-sensitivity C-reactive protein; IL-6, interleukin-6; NAD(P)H, reduced nicotinamide adenine dinucleotide (phosphate); NF-κB, nuclear factor κB; PKA, protein kinase A; RAGE, receptor for advanced glycation end-products; RCT, randomized controlled trial; ROS, reactive oxygen species; T2D, type 2 diabetes; TGF-β, transforming growth factor β; TNF-α, tumor necrosis factor-α.

## Modulation of the RAAS

There is increasing evidence that links GLP-1 receptor agonism to RAAS modulation. Clinical observations following GLP-1 infusion and single-dose liraglutide administration showed reduced circulating angiotensin II in both healthy individuals and patients with T2D [73,74]. While the precise mode of action remains under active investigation, different pathways may account for this reduction in circulating angiotensin II [75]. GLP-1 RAs may suppress renin secretion, as attenuated proximal tubular sodium reabsorption increases sodium delivery to the macula densa, thereby activating tubuloglomerular feedback [76]. In addition, GLP-1 may down-regulate tissue-level angiotensin II production, given that chronic GLP-1 RA administration was associated with decreased renal cortical angiotensin II content and urinary angiotensinogen excretion, whereas GLP-1 receptor blockade produced the opposite response [77]. This modulation of the RAAS—and the reduction of potent vasoconstrictors like endothelin—likely contributes to the stability of the glomerular filtration barrier, while suggesting that GLP-1 RAs confer nephroprotection independently of systemic effects on glycemic control or weight loss [78–80].

## Attenuation of oxidative stress and chronic endothelial inflammation

Experimental studies have implicated several mechanisms, including the induction of natriuresis and the inhibition of oxidative stress, inflammation, and fibrosis. Murine studies have indicated that GLP-1 RAs protect against renal oxidative stress by activating the cyclic adenosine monophosphate (cAMP)-protein kinase A (PKA) pathway and by inhibiting NAD(P)H oxidase, potentially reducing ROS levels in glomeruli and tubules [81]. Nuclear factor erythroid 2-related factor 2 (Nrf2), a key regulator of redox balance and antioxidant gene expression, is implicated in various oxidative stress-related diseases, and GLP-1 RAs may have protective effects against oxidative stress by enhancing antioxidant enzyme expression and activating the Nrf2 signaling pathway [82,83]. Translational evidence further implicates mitochondrial biology as a potential mediator of GLP-1-induced organ protection. In cultured tubular epithelial cells, GLP-1 RA exposure was associated with preserved mitochondrial membrane potential, reduced ROS generation, and attenuated mitochondrial apoptosis [84]. In addition, reduced ROS production and improved mitochondrial membrane potential in leukocytes of GLP-1 RA-treated patients with T2D were reported [85].

In streptozotocin-induced diabetic rats, liraglutide was associated with improvements in markers of oxidative stress and lower albuminuria, without significantly altering plasma glucose or body weight [86]. Consistently, GLP-1 RAs appeared to attenuate kidney injury through anti-inflammatory actions in a rat model of type 1 diabetes, again without lowering blood glucose [87]. Direct, non-metabolic renoprotection is further supported by studies using the nephrotoxic serum nephritis model, which, by lacking confounders of the metabolic syndrome such as hyperglycemia and obesity, allows for a better evaluation of the intrinsic renoprotective capacity of GLP-1 RAs [88]. In this model, liraglutide was reported to improve GFR, reduce albuminuria, and attenuate renal fibrosis. Correspondingly, a modulation of gene expression pathways involved in inflammation and fibrosis was observed [80].

Evidence from mouse models suggests that GLP-1 RAs, such as semaglutide, may exert anti-atherosclerotic effects by reducing arterial plaque formation and down-regulating aortic inflammatory pathways, potentially independent of weight loss or lipid lowering [89]. Correspondingly, meta-analyses of RCTs have reported that GLP-1 RAs reduce circulating inflammatory markers, including C-reactive protein (CRP) and IL-6 [90,91]. Consistent with this, an exploratory patient-level analysis pooling semaglutide trials (SUSTAIN 3 and PIONEER 1, 2, and 5) reported a reduction in high-sensitivity CRP versus comparators in patients with T2D, although the reduction was not significant in the trial conducted in participants with CKD (PIONEER 5) [92].

## Direct cellular and molecular mechanisms of GLP-1 RA-mediated nephroprotection

GLP-1 RA actions on monocytes, macrophages, endothelial cells, and vascular smooth muscle cells suggest a further potential anti-atherosclerotic effect [93–96]. Advanced glycation end products, which accumulate with age, diabetes, and renal failure, promote vascular inflammation via their receptors (RAGE). GLP-1 may reduce this inflammation by down-regulating RAGE expression through cAMP signaling [97]. Furthermore, GLP-1 and its analogs may exert immunomodulatory effects by influencing macrophage polarization, promoting the anti-inflammatory M2 phenotype over the pro-inflammatory M1 phenotype [98,99]. Treatment with dulaglutide was associated with lower serum type VI collagen formation and higher urine type III collagen degradation than insulin glargine, suggesting a potential beneficial effect on renal fibrosis [100]. Mechanistically, GLP-1 RAs may inhibit fibrotic responses by suppressing the TGF-β pathway, which plays a central role in the development and progression of kidney fibrosis [87,101–103]. Furthermore, GLP-1 and its analogs might have direct effects on podocyte protection, a key component of the glomerular filtration barrier, by modulating several crucial signaling pathways, including the pro-inflammatory cytokine TNF-α and its downstream transcription factor, nuclear factor kappa B (NF-κB) [104–107].

The results of the mechanistic REMODEL trial (NCT04865770) will further increase the understanding of the renal mechanism of action of semaglutide [108]. In addition, several subsequent mechanistic studies across diverse indications have been initiated, including tirzepatide for CKD and overweight or obesity, semaglutide for type 1 diabetes, and retatrutide for renal function in participants with overweight or obesity and CKD with or without T2D [109].

## Dose dependency of potential cardiovascular and kidney benefits

As most cardiovascular outcome trials employed a single maintenance dose or pooled their dose arms for the primary analysis, evidence on whether the cardiovascular and kidney benefits of GLP-1 RAs are dose-dependent remains limited. Although the trial was not powered for a head-to-head comparison of different doses, randomized dose-comparison data are available from AMPLITUDE-O, in which participants were randomized to receive placebo, 4 mg, or 6 mg efpeglenatide weekly, and a dose-response relationship was assessed using the log-rank test and χ^2^ statistic for trend [110]. A graded effect across these arms was shown for both MACE and a composite kidney outcome [110]. An analogous dose dependency has been described for changes in albuminuria with tirzepatide: in a pooled analysis of SURPASS-1–5, UACR reductions increased progressively across the 5, 10, and 15 mg doses, though mediation analyses suggested that approximately half of the albuminuria lowering was attributable to weight loss [111]. Within STEP 2, UACR changes were −14.8% with 1.0 mg semaglutide and −20.6% with 2.4 mg semaglutide [41]. However, in the AWARD-7 trial, both 0.75 mg and 1.5 mg weekly dulaglutide doses attenuated the decline in cystatin C–based eGFR relative to insulin glargine to a similar degree [37]. While these limited and in part discordant data leave open whether the cardiovascular and kidney protective effects of GLP-1 RAs are dose-dependent, adverse gastrointestinal events, most notably nausea, appear to potentially follow a similar dose-dependent pattern.

## Adverse events and long-term safety considerations of GLP-1 RA

While the expression of GLP-1 receptors across various organ systems has raised questions about potential long-term risks, empirical data from 20 years of clinical use suggest that most associated adverse events (AEs) are mild in nature. Their clinical use is frequently accompanied by specific gastrointestinal AEs, most notably nausea, diarrhea, and vomiting [46]. These symptoms occur in up to 50% of patients, typically during the dose-escalation phase [69]. While generally mild-to-moderate and transient—resolving within 4–8 weeks—gastrointestinal distress can be dose-limiting and contributes to discontinuation rates of approximately 12% in phase 3 clinical trials [69]. Furthermore, severe or protracted symptoms may compromise oral intake, potentially leading to volume depletion and subsequent acute kidney injury [112]. The risk of hypoglycemia is overall low, but increases when GLP-1 RAs are used in combination with insulin or sulfonylureas [113,114]. More serious, though less frequent, risks include pancreatitis and, in rare cases, pancreatic cancer, although the causal relationship remains debated, and analyses of both trial-based and longitudinal real-world datasets show an unclear safety signal regarding pancreatic AEs [112,115,116]. Although overall thromboembolic risk appears stable, long-term GLP-1 RA use has been linked to a significantly higher incidence of deep vein thrombosis, with approximately 25 (5–52) more events per 10 000 person‐years [117]. This potential risk highlights the need for vigilant monitoring during extended treatment periods and within cardiovascular outcome trials. One CVOT reported an increase in retinopathy probably attributed to rapid glycemic improvement. Pending results from the FOCUS trial in 2027, baseline retinopathy screening and individualized risk-benefit discussions are recommended prior to treatment initiation [46,118]. Thyroid C-cell hyperplasia and an increased risk of medullary thyroid carcinoma have been observed in rodent studies, but these findings have not been definitively replicated in humans [119].

## Renal and musculoskeletal implications of GLP-1 RA-induced weight reduction

While GLP-1 RA-induced weight loss has raised concerns regarding lean body mass (LBM) attrition, evidence from the STEP 1 trial indicates that, although total LBM decreased in absolute terms, the proportion of LBM relative to total body mass increased during treatment [120]. Although high-protein, carbohydrate-restricted diets are suggested to preserve muscle mass, these strategies require caution in patients with CKD due to risks of glomerular hyperfiltration or ketosis [121]. The clinical intersection of muscle mass and renal function is particularly relevant when monitoring GFR. Serum creatinine is strongly influenced by skeletal muscle mass, whereas cystatin C is largely muscle-independent but is affected by adiposity and inflammation; consequently, the marked shifts in body composition during substantial weight loss might bias both creatinine- and cystatin C-based eGFR. To address this, the SMART trial utilized iohexol clearance to provide a direct measure of GFR (mGFR), reporting that 24 weeks of semaglutide treatment and a 10 kg weight loss did not significantly alter mGFR or creatinine- or cystatin C-based eGFR. These results are consistent with findings from the FLOW, AWARD-7, and SURPASS-4 trials, which collectively suggest that eGFR remains a reliable metric despite significant treatment-induced changes in body composition [37,52,54].

While weight loss does not appear to correlate with GFR changes, it is positively associated with reductions in the UACR [43]. This antiproteinuric effect appears to be independent of systemic hemodynamic changes, possibly reflecting improved endothelial glycocalyx integrity secondary to reduced adiposity or a direct reduction in glomerular hypertension [122].

## Clinical efficacy and safety of combination therapy in patients with renal disease

Current clinical guidelines recognize GLP-1 RAs as foundational for managing patients with T2D and concomitant CKD. The 2025 American Diabetes Association Standards of Care prioritize GLP-1 RAs and SGLT2is to attenuate CV and renal risk. GLP-1 RAs are preferentially recommended to retard kidney disease progression and mitigate CV events, and in advanced renal impairment (eGFR <30 ml/min/1.73 m^2^), GLP-1 RAs offer a favorable safety profile compared with insulin due to diminished hypoglycemic risk and sustained renoprotection [123]. Combination therapy is emerging as a new standard for diabetic and non-diabetic CKD, and GLP-1 RAs may constitute a fourth pillar alongside RAAS inhibitors, SGLT2is, and finerenone [124]. The scarcity of dedicated trials specifically powered to evaluate new combination therapy limits definitive conclusions regarding long-term outcomes [69]. Therefore, pooled analyses of RCTs are frequently used to evaluate the effect of combination therapy. In pooled data from eight RCTs, including 1895 patients with T2D, dual therapy of GLP-1 RAs and SGLT2is yields superior reductions in HbA1c, systolic blood pressure, and body weight compared with monotherapy [125]. The FLOW trial indicated that the efficacy of semaglutide in modulating cardiorenal outcomes remained consistent irrespective of the concomitant administration of SGLT2i. This finding was mirrored in a meta-analysis of 12 cardiovascular outcome trials, which showed that SGLT2i efficacy is not attenuated by concomitant GLP-1 RA therapy, thus suggesting independent effects of these therapies [126]. Furthermore, evidence from real-world datasets corroborates the superior cardiovascular protection afforded by dual therapy as, compared with other glucose-lowering regimens, concomitant use of GLP-1 RAs and SGLT2i was associated with a lower incidence of heart failure (HF) and major adverse cardiac and cerebrovascular events [127].

Strategic combination therapy may also optimize safety profiles; for instance, similar to observations with SGLT2i, the use of GLP-1 RAs has been associated with a reduced incidence of hyperkalemia. This effect might facilitate the prolonged use of RAAS inhibition, as evidenced by a large cohort study where GLP-1 RA treatment resulted in lower hyperkalemia rates and extended RAAS inhibition persistence compared with dipeptidyl peptidase-4 inhibitors [128,129].

## Conclusion

The therapeutic landscape of CKD has undergone a significant paradigm shift, with GLP-1 RAs emerging as a cornerstone of cardiorenal protection. Beyond their established role in glycemic management, evidence from landmark trials—most notably the FLOW and SELECT studies—demonstrates that GLP-1 RAs can substantially mitigate the risk of MACE and slow the progression of diabetic kidney disease. These benefits appear to be mediated through pleiotropic mechanisms, including the attenuation of oxidative stress, inflammation, and fibrotic signaling, which extend beyond metabolic improvements. While gastrointestinal side effects remain a primary consideration for adherence, the overall safety profile appears to be favorable in the majority of patients, particularly regarding the low risk of hypoglycemia in advanced renal impairment. As clinical guidelines increasingly prioritize dual therapy with SGLT2i, GLP-1 RAs are poised to become a “fourth pillar” of diabetic CKD management alongside RAAS inhibitors, SGLT2i, and mineralocorticoid receptor antagonists. While the clinical utility of GLP-1 RAs in non-diabetic CKD remains to be fully elucidated, their pharmacological profile suggests that their effects may be additive to those of current therapies. Further research into dedicated combination trials will be essential to fully optimize thesecombination strategies and further reduce the residual cardiorenal risk in both diabetic and non-diabetic populations.

### Search strategy and selection criteria

A comprehensive literature search was conducted across PubMed, Google Scholar, MEDLINE, and the Cochrane Library for English-language abstracts and full-text articles published up to March 2026. The search focused on the implications of GLP-1-based therapeutics, novel combinations in the context of CKD, and the underlying mechanisms mediated by GLP-1 RAs. Search terms included, but were not limited to, “incretin-based therapy”, “GLP-1 receptor agonist”, “glucagon-like peptide-1”, “glucagon”, “dual agonist”, “nephroprotection”, “cardioprotection”, “kidney disease”, “cardiovascular disease”, “diabetic nephropathy”, and “type 2 diabetes”, utilized both independently and in combinations. Reference lists from original research, clinical guidelines, and existing (systematic) reviews or meta-analyses were manually screened.

## Open Access

Open access for this article was enabled by the participation of Newcastle University in an all-inclusive Read & Publish agreement with Portland Press and the Biochemical Society under an agreement with JISC.

## Abbreviations

AEs: adverse events
BMI: body mass index
cAMP: cyclic adenosine monophosphate
CI: confidence interval
CKD: chronic kidney disease
CRP: C-reactive protein
CV: cardiovascular
CVD: cardiovascular disease
eGFR: estimated glomerular filtration rate
GFR: glomerular filtration rate
GLP-1 RA: glucagon-like peptide-1 receptor agonist
HbA1c: glycated hemoglobin
HF: heart failure
HR: hazard ratio
IL-6: interleukin-6
LBM: lean body mass
MACE: major adverse cardiovascular events
NAD(P)H: reduced nicotinamide adenine dinucleotide (phosphate)
NF-κB: nuclear factor kappa B
Nrf2: nuclear factor erythroid 2-related factor 2
PKA: protein kinase A
RAAS: renin-angiotensin-aldosterone system
RAGE: receptor for advanced glycation end-products
RCT: randomized controlled trial
ROS: reactive oxygen species
SAEs: serious adverse events
SGLT2i: sodium-glucose cotransporter 2 inhibitors
T2D: type 2 diabetes
TGF-β: transforming growth factor-β
TNF-α: tumor necrosis factor-α
UACR: urinary albumin-to-creatinine ratio
